# Human Milk, More Than Simple Nourishment

**DOI:** 10.3390/children8100863

**Published:** 2021-09-28

**Authors:** Giulia Vizzari, Daniela Morniroli, Federica Ceroni, Elvira Verduci, Alessandra Consales, Lorenzo Colombo, Jacopo Cerasani, Fabio Mosca, Maria Lorella Giannì

**Affiliations:** 1 Department of Clinical Sciences and Community Health, University of Milan, Via Commenda 19, 20122 Milan, Italy; giulia.vizzari@unimi.it (G.V.); daniela.morniroli@unimi.it (D.M.); federica.ceroni@unimi.it (F.C.); alessandra.consales@unimi.it (A.C.); jacopo.cerasani@unimi.it (J.C.); fabio.mosca@unimi.it (F.M.); 2 Department of Pediatrics, Vittore Buzzi Children’s Hospital, University of Milan, 20154 Milan, Italy; elvira.verduci@unimi.it; 3 Department of Health Sciences, University of Milan, 20154 Milan, Italy; 4 Fondazione IRCCS Ca’ Granda Ospedale Maggiore Policlinico NICU, Via Commenda 12, 20122 Milan, Italy; lorenzo.colombo@policlinico.mi.it

**Keywords:** breast-milk-derived stem cells, bio-factors, growth factors, health outcomes, microbiome, human milk oligosaccharides

## Abstract

Human breast milk not only has nutritional properties but also holds a functional role. It contains various bioactive factors (lactoferrin, lysozyme, leukocytes, immunoglobulins, cytokines, hormones, human milk oligosaccharides, microbiome, microRNAs and stem cells) shown to contribute to several short- and long-term health outcomes. Some of these factors appear to be involved in the infant’s neuro-cognitive development, anti-oncogenic processes, cellular communication and differentiation. Furthermore, breast milk is increasingly recognized to have dynamic characteristics and to play a fundamental role in the cross-talking mother-neonate. This narrative review aims to provide a summary and an update on these bioactive substances, exploring their functions mainly on immunomodulation, microbiome and virome development. Although the knowledge about breast milk potentiality has significantly improved, leading to discovering unexpected functions, the exact mechanisms with which breast milk exercises its bioactivity have not been completely clarified. This can represent a fertile ground for exploring and understanding the complexity behind these functional elements to develop new therapeutic strategies.

## 1. Introduction

The nutritional value of breast milk has been widely recognized and discussed in the literature [1]. Its composition in terms of macro and micronutrients has been widely investigated, highlighting how these components change dynamically according to factors including lactation stages, time of the day, maternal and infants’ characteristics [2]. Breastmilk contains all the nutrients the infant needs to provide the best opportunities for growth and development. For these reasons, in accordance with the World Health Organization (WHO) [3], the American Academy of Pediatrics (AAP) [4] and the European Society for Pediatric Gastroenterology, Hepatology and Nutrition (ESPGHAN) [2], human milk is the physiological and unequalled method for feeding infants at least up to the first six months of life and later, along with complementary foods for up to two years of age or beyond.

A considerable amount of evidence indicates that breast milk cannot be considered simple nourishment but represents a significant predictor for the future of both lactating mother and newborn [5]. Furthermore, through biochemical and immunomodulating signals, breast milk allows a specific communication between mother and child to promote human well-being and the infant’s growth and development [5,6]. Breastmilk represents a link in mother-infant signaling thanks to the provision of several bioactive factors such as lactoferrin, lysozyme, leukocytes, immunoglobulins, cytokines, hormones, stem cells, human milk oligosaccharides (HMOs), microbiota, microRNAs, antioxidants and growth factors. From an evolutionary point of view, the transmission of human milk’s bioactive factors from mother to offspring represents a resource for future health outcomes involved in developing the infant’s cognitive and immune system and gut microbiological colonization and maturation. Accordingly, several epidemiological studies have supported the protective effect of human milk against infections both in the short and long term, such as pediatric otitis media, gastroenteritis and respiratory tract infections, as well as its crucial contribution in reducing the risk of developing cardiovascular diseases, obesity and type 2 diabetes [7,8,9,10].

The function of breast milk, however, is also to promote the adaptation of the newborn to the surrounding environment, not only by protecting it from infectious or non-communicable diseases, but also by helping the development of many body functions, including the digestive function, thanks to the presence of gastrointestinal hormones and peptides [11]. In addition, breast milk promotes the establishment of the circadian rhythm. It is now known that breast milk varies throughout the day as nutritional components and as amino acids and hormones (tryptophan and melatonin), which helps the infant to regulate its still immature circadian rhythm [12]. Although these multiple benefits associated with breastfeeding have been extensively investigated, the synergistic mechanisms by which breast milk is responsible for these short- and long-term health outcomes have not yet been completely understood.

This narrative review aims to provide a summary and an update on these bioactive substances, exploring their functions and several impacts in terms of immunomodulation, microbiome and virome development. From April to June 2021, we searched the PubMed database for randomized controlled trials, cohort studies, systematic reviews and meta-analyses relating to breast milk using specific keywords such as breast milk-derived stem cells, bio-factors, growth factors, health outcomes, microbiome, and human milk oligosaccharides. Preference was given to the sources published within the past five years, and preclinical studies were excluded.

## 2. Immune Health

**Figure 1:**
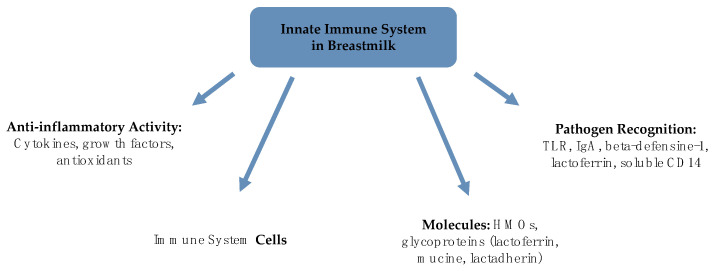
Innate immune system in human milk (HM).

Recent literature has well established that breast milk can be considered a complex innate immune system that modifies its characteristics concerning the timing of delivery, lactation stage, and mother and infant’s health status [9]. Moreover, the mammary gland’s nutritional and immune functions seem to have a concomitant development [13]. Breast milk should be thought of as a co-adapting system, in a closely connected mother-breast milk-infant triad. Every variation in one of these three components could affect maternal health or infant development [5]. Breastfeeding allows the transmission of immunomodulating factors which contribute to immediate and direct protection from infections and the development of the newborn’s immune system and intestinal microbiota (Figure 1).

It is widely known that breast milk contains numerous immune functional proteins through which the mother confers passive immunity to the newborn. All types of immunoglobulins (Igs) are present in breast milk. However, IgAs are the predominant one, followed by IgGs. Their concentration decreases in the first 12 weeks after birth, in accordance with an increase in the newborn’s own production [14,15]. In addition to providing a passive immunity, breast milk IgAs bind to gut bacteria, hindering their overgrowth, inhibiting pathogens’ attachment to the gastrointestinal mucosal surface and neutralizing microbial toxins, thus conferring an antimicrobial defense to the neonatal gastrointestinal tract [16].

The literature has highlighted a possible role played by breast milk IgAs in modifying the infants’ microbiota. According to a recent study published in 2019, the lack of IgAs-bacteria binding could be responsible for the increased proliferation of anaerobic bacteria, causing inflammatory diseases such as necrotizing enterocolitis (NEC) [17]. In line with these results, Gopalakrishna et al. showed that low IgAs concentration in the breast milk of preterm infants was associated with insufficient microbiome diversity, which may represent an additional risk factor for the development of NEC [18]. The importance of IgAs concentration in preventing NEC was confirmed in a mouse model of NEC in which, even if breastfed, pups raised by IgA-deficient mothers had a higher risk of developing NEC [18,19].

Interestingly, some studies have hypothesized an even closer bidirectional communication from the newborn to the mother. In fact, due to a retrograde flow of milk, pathogens present in the infant’s mouth could reach the mammary gland, triggering the maternal production of antibodies in order to protect the infant and to support its immature immune system [20].

According to recent studies, maternal health status influences the concentration of immunoglobulins in breast milk. In particular, IgA levels have been reported to be lower in the mature milk of mothers with gestational diabetes, post-partum stress, anxiety and depression [14]. Furthermore, high levels of IgA, cytokines, oligosaccharides and other immune factors in human milk are associated with a lower risk of food allergy during childhood [15,16]. In their study, Joseph et al. showed that lower allergy prevalence was associated with higher transforming growth factor beta (TGF-β) levels in breast milk [21].

During the SARS-CoV-2 (severe acute respiratory syndrome coronavirus-2) pandemic, much concern has been generated about the possibility of the virus being transmitted during breastfeeding [22]. However, recent studies have shown that viral genome detection in breast milk is uncommon. In particular, Pace et al. in their cohort study evaluated several human milk samples from 18 women following COVID-19 diagnosis without ever detecting SARS-CoV-2 RNA [23]. A recent meta-analysis has concluded that SARS-CoV-2 genome presence in breast milk is uncommon, whilst anti-SARS-CoV-2 antibodies are a common finding [24]. In their study, Pace et al. demonstrated the passage through breast milk of anti-SARS-CoV-2 IgAs and IgGs with neutralizing effects against SARS-CoV-2 [23,25]. These results confirm the immunological advantage of breastfed infants and support the recommendations to continue breastfeeding during mild-to-moderate maternal COVID-19 illness [23,24]. In addition, recent studies confirmed that anti-SARS-CoV-2 antibodies can be found in lactating vaccinated women, confirming an immune transfer to neonates occurring via breast milk [26].

Most recent studies highlighted the presence of Toll-like receptors (TLR), soluble CD14 and human Beta-defensin-1 in breast milk, which, along with inflammatory mediators such as lysozyme, lactoferrin, alpha-lactalbumin and osteopontin, would be able to induce an active immune response against pathogens in the newborn [27]. Moreover, literature has well established that the premature infant’s gut has an elevated expression and activity of Toll-like receptor-4 (TLR4) belonging to a family of bacterial recognition receptors, causing an abnormal inflammatory response that leads to the development of NEC [28]. Human milk acts as a powerful TLR4 inhibitor thanks to IgA, microbiota, nondigestible oligosaccharides and some growth factors such as the epidermal one (EGF), which directly inhibits TLR4 signaling [28,29].

**Figure 2:**
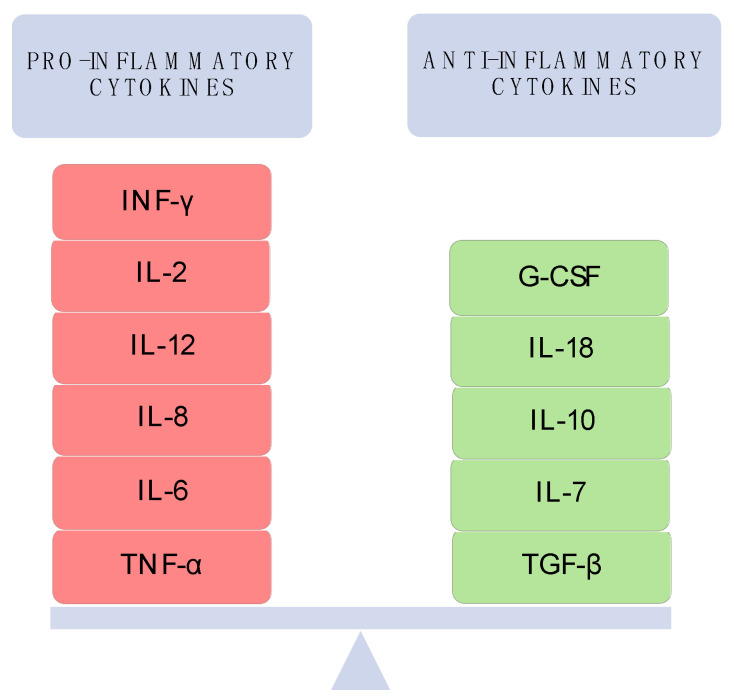
Cytokines in breast milk. (Modified from Kielbasa et al., 2021.)

Cytokines are small bioactive molecules that can modulate the body’s immune response by participating in the complex signaling between immune cells. All types of cells produce cytokines. These molecules are characterized by synergism in action, antagonism and aggregation activity. They can oppose the action of one cytokine or induct another cytokine and have a precise regulation through autoregulation-stimulation or inhibition of their own production [11]. The predominance of pro-inflammatory cytokines causes a systemic inflammatory response, promoting the body’s defenses when its homeostasis is disrupted. Cytokines in human milk are essential for the development of the immune system in newborns. Breast milk is the primary source of cytokines, particularly anti-inflammatory cytokines. This is a paramount property of human milk, considering that newborns are commonly deficient. Pro- and anti-inflammatory cytokines in mothers’ milk are presented in Figure 2 [30].

Recent studies underline the importance of human milk lactoferrin as immunomodulating factor. Lactoferrin is an iron-binding glycoprotein with high concentrations in colostrum, declining then throughout lactation [31]. Binding iron, lactoferrin plays a crucial role in preventing pathogens’ growth and proliferation, presents a direct cytotoxic effect against bacteria, viruses, and fungi, and promotes the growth of probiotic bacteria [32]. As lactoferrin can enhance the host’s immune defenses, it has been postulated that it can contribute to prevent sepsis. However, more studies are needed to confirm this function [33]. In addition, although the literature has clarified the anti-inflammatory action of lactoferrin by blocking many pro-inflammatory cytokines, its protective role against the development of NEC has been questioned [33]. Griffith et al. in their recent large randomized control trial of 2203 preterm infants showed that lactoferrin supplementation did not decrease the incidence of NEC or infections [34].

In addition, immune system cells such as leukocytes represent another important bioactive component of human milk. Contrary to what was thought in the past, leukocytes are part of the immune inheritance of the mother-breast milk-infant triad. As recent studies show, the leukocyte content of breast milk is not fixed but presents higher concentrations in colostrum and during the infant’s or mother’s infections, highlighting even more the role of the mother’s milk as a means of communication between the dyad in adapting to the environment [35].

## 3. Microbiome

Recent literature has defined breast milk as the first probiotic food in the entire life of a human [36]. More than 200 bacterial species in human milk contribute to the colonization of the newborn’s gastrointestinal tract and modulation of the infant’s microbiota development [37]. The most common bacterial species identified in breast milk were *Streptococcus* and *Staphylococcuss*, followed by *Bifidobacterium*, *Lactobacillus*, *Propionibacteria*, *Enterococcus* and *Enterobacteriaceae* (Table 1) [38,39].

Pannaraj et al. in a recent longitudinal study demonstrated that the bacterial component present in exclusively breastfed infants’ feces presented an overlap of about 28% with the bacterial component of mother’s milk and an overlap of about 10% with the bacterial component of maternal areolar skin [39]. Thus, overall, breast milk could provide about 25% of the intestinal microbiota of an exclusively breastfed infant at one month of life. In fact, a breastfed infant is exposed to approximately over 700 species of bacteria per day [39].

A recent study by Bäckhed et al. highlighted that formula-fed infants develop a gut microbiota more enriched in Clostridia species than breast-fed babies, which present more Bifidobacterium and Lactobacillus species in their gut microbiota (Table 1) [40]. This evidence suggested a relevant contribution played by breast milk in the correct development of the immune system which, eventually, is connected with better future health outcomes. Emerging evidence has demonstrated that, through breast milk, the mammary gland supplies not only for the bacterial part of the microbiome but also for the viral one, called the virome [16]. The human milk virome includes eukaryotic viruses, bacteriophages and other viral particles which are considered safe and favorable to the neonate’s health, whereas they seem to inhibit the transmission of pathogenic viral strains [41]. With particular regards to bacteriophages, the virome could promote the development of beneficial bacteria for the newborn and eliminate detrimental ones, thus modeling the microbiome [42].

Furthermore, researchers focused their attention on how the colonization of breast milk occurs. This phenomenon is partly explained by the migration of bacteria into breast milk from the bacterial flora of the maternal areolar skin and infant’s mouth. In addition, some recent studies focused on the existence of an entero-mammary pathway that occurs during late pregnancy and lactation and by which maternal gut bacteria could reach the mammary gland through the bloodstream, involving gut monocytes as a vehicle [38]. Some studies, however, have also shown a possible translocation of bacteria from the mother’s oral cavity to the mammary gland and then to the breast milk. Maternal oral bacteria and milk microbiota are partially overlapping, supporting the theory of an oro-mammary translocation [43]. Carrothers et al., in a recent study, showed that infant gut microbiota could be additionally influenced by maternal diet during the perinatal period. This hypothesis might explain the presence of specific breast milk bacteria found neither on the mother’s skin nor in the infants’ mouth [44]. According to recent studies, maternal mental health status and postnatal psychosocial distress could impact the breast milk microbiota. Browne et al. showed a negative correlation between breast milk microbiota diversity and maternal psychosocial distress at 3 months postpartum [45]. According to these findings, maternal psychosocial distress and maternal health status may affect infants’ development and future health. Considering mother, breast milk and infant as the components of a connected triad within a “life-cycle” perspective, we should take care of women’s health to improve their offspring’s health [46,47].

This wide biodiversity in the breast milk microbiota depends on various factors. Recent studies highlighted a higher variability in the human milk microbiota composition of mothers who gave birth spontaneously compared to those who underwent a cesarean section. Differences in human milk microbiota were also found in mothers who received elective cesarean section versus mothers who received non-elective cesarean section [48,49]. Moreover, one of the main factors affecting breast milk microbiota is the administration of antibiotics to mothers in the peripartum [50]. On the other hand, no significant differences were found in breast milk microbiota based on maternal age or infant gender [51,52]. 

Because of the significant risk of infection and adverse short- or long-term outcomes, breast milk microbiota plays an even more beneficial role in preterm infants, especially infants with very low birth weight [53]. A recent longitudinal study conducted by Biagi et al. analyzed how the human milk microbiota composition changed among a cohort of moderately preterm infants. According to Biagi et al., infants latching directly to the mother’s breast could modify breast milk microbiota composition with a high prevalence of bifidobacterium, which represents a typical oral microbe [54]. Therefore, it could be hypothesized that direct breastfeeding and skin-to-skin practice represent an independent protective effect of breast milk, helping the health-promoting features of the infant gut microbiome [55]. For this reason, direct breastfeeding should be started as soon as possible among mothers of preterm newborns [55]. 

On the other hand, sometimes maternal human milk may not be available, so donor human milk represents an optimal nutritional solution for preterm infants [55]. Current guidelines recommend the pasteurization of human milk banks to inactivate viral and bacterial agents [56]. However, even if macronutrients are preserved by heat treatment, some bioactive beneficial factors could be partly compromised. Infants fed with donor milk present a significantly higher abundance of Staphylococcaceae, Clostridiaceae and Pasteurellaceae and lower Bifidobacteriaceae than breastfed infants [33]. This effect represents one of the weakest points of donor milk [57]. For this reason, recent literature has focused on improving milk processing techniques to minimize the risk of infection and preserve the human milk bioactivity as much as possible [58].

Different factors resulting in a less diverse and more “dysbiotic” gut microbiota would seem partly responsible for a suboptimal stimulation of the immune system development during infancy. Infants who develop asthma later in life and remain healthy present different microbiota patterns [29]. Supporting these findings, Dogaru et al. found a lower risk of asthma in breastfed infants than in formula-fed ones [59]. In addition, two recent longitudinal studies reported a reduced risk of wheezing and asthma among breast-milk-fed infants, and this correlation seems dose-dependent and more relevant in directly breastfed children than those fed with expressed breast milk [60,61]. However, more studies are needed to evaluate if this latter finding could be due to the alteration of human milk bioactive compounds such as oligosaccharides or microbiota during the pumping and storage process [60,62]. How the milk microbiota is modulated is a matter of debate, and many studies are still needed to lead to a comprehensive understanding.

## 4. Human Milk Oligosaccharides

Human milk oligosaccharides (HMOs) are the third most abundant component of human milk. HMOs do not provide direct nutritional value to the infant but play a key role in modulating the development of the infant’s gut microbiota and immune system [63]. HMOs seem to reduce pathogens’ adhesion to the host mucosal surface, offering themselves as an alternative target to viruses or bacteria [64]. Moreover, HMOs promote the barrier function of the gastrointestinal tract and modulate the expression of intestinal epithelial genes involved in the regulation of the immune system [51].

Following the known capacity of HMOs to reduce viral adhesion to the host mucosal surface, the hypothesis of a possible protective role of HMOs in preventing, limiting or modulating SARS-CoV-2 infection has been formulated even if not explored yet, as sialic acids present on the cell surface may be recognized by this virus as additional receptors for the binding site [65].

Their main feature is represented by their extreme qualitative and quantitative variability. In fact, HMO concentration in breast milk varies from 1–10 g/L in mature milk to 15–23 g/L in colostrum [63]. From a qualitative point of view, HMOs composition appears to be highly influenced by maternal genotype. Oligosaccharides are complex glycans composed of lactose, elongated at the reducing end with fucosylated groups or sialyl-N-acetyl-lactosamine units. In mature milk, 35–50% of HMOs are fucosylated, 12–14% are sialylated and 42–55% are non-fucosylated neutral ones [66]. Recent literature highlighted that different HMO structure corresponds to different HMO functions. Moreover, the profile of HMOs varies between women depending on genetic features but remains substantially constant throughout lactation for the same mother [67]. These different HMO profile clusters are also called HMO lactotypes [63]. Moreover, the presence of a homozygous genotype for an inactivating mutation on the fucosyltransferases 2 (FUT 2) gene can lead to enzyme inactivation, resulting in mothers with a non-secretory status, distinguished from secretor mothers, who present an active copy of the FUT2 gene. Similarly, the presence of an inactivating FUT 3 gene mutations are specific to “Lewis negative” mothers, as opposed to Lewis positive ones, when FUT3 is active [68].

Much remains to be discovered regarding HMOs. These molecules seem to be present in maternal systemic circulation from the very beginning of gestation, before the onset of lactation, probably originating from an early synthetic activity of the mammary gland. According to the recent literature, HMOs could contribute to the adaptation processes of the immune system and the maternal metabolism during pregnancy, playing a protective role against both infections and immune disorders related to pregnancy. Their concentration in the maternal serum is significantly lower than that calculated in breast milk [69]. However, a recent study found that HMOs with similar composition were detected in amniotic fluid, breast milk and maternal serum and urine samples, indicating infant exposure to specific HMOs since fetal life, with potential implications for fetal well-being and long-term health outcomes [70]. A study by Austin et al. compared the HMO content of 500 breast milk samples from mothers of preterm and term infants and observed higher concentrations of sialylated HMOs in the preterm milk. These differences were partly explained by a reduced activity of fucosyltransferase-2 enzyme in mothers of infants born prematurely during the first month of breastfeeding, resulting in a reduced concentration of fucosylated HMOs [71].

A recent study, using an animal model, showed an improved hippocampal synaptic activity after administration of 2-fucosyllactose and demonstrated how HMOs were involved in modulating the brain-gut axis, allowing the communication between the central nervous system and the intestine [72,73]. Saben et al. examined the relationship between maternal glucose homeostasis and the composition of HMOs from 136 healthy women and found a strong correlation between maternal insulin sensitivity and difucosyllactose concentrations in breast milk of secretor mothers. In fact, secretor mothers present lower insulin sensitivity and a more significant amount of sialylated HMOs than non-secretory ones. These findings support a protective and growth-promoting role for sialylated HMOs [74,75]. In light of the recent evidence indicating that rotaviruses appear to have learned to use HMOs to their advantage, the potential role of HMO in increasing the efficacy of rotavirus vaccines has been explored. In particular, Ramani et al. detected a greater efficacy of the rotavirus vaccine when associated with specific HMOs, thus introducing the possibility of implementing new vaccination strategies [76].

## 5. Stem Cells

A recent discovery among bioactive factors of breast milk is the presence of a heterogeneous population of stem cells, which seems to be characterized by immune privilege and non-tumorigenic properties [38]. Breast milk stem cells are pluripotent lines, thus being able to become specific breast epithelial cells and differentiate in other cellular types, including neural-like ones [39]. Hosseini et al. demonstrated that the exposure of breast milk stem cells to neurogenic stimuli in vitro led to the differentiation into all the three neural lines: neurons, oligodendrocytes and astrocytes [39]. Moreover, because both the mammary gland and the nervous system share the exact embryonic origin, it might be postulated that breast milk stem cells contribute to the development of the enteric nervous system. This supposition could partly explain why non-breast-fed preterm infants showed a significantly higher risk of developing necrotizing enterocolitis [40,41]. Experimental studies, using animal models, have shown that stem cells present in breast milk could reach the systemic bloodstream of the offspring through gastrointestinal infant absorption, thus reaching some target organs such as central nervous system, thymus, pancreas, spleen and kidney, where they integrate and differentiate into functional cells [42]. Moreover, as recent studies highlighted, both colostrum and transitional human milk are an abundant reservoir of hematopoietic stem/progenitor-like cells [77].

In a recent pilot study, the cellular components of colostrum and mature milk of preterm babies were compared using flowcytometry. The authors found that preterm mature milk has higher expressions of hematopoietic stem cells, mesenchymal stem-like cells, immune cells, some cell adhesion molecules and side-population cells than colostrum. Even if more studies are needed to support this pilot finding, a higher cell content in mature milk of preterm babies seems important in future health and growth outcomes [78]. Breast milk stem cells could therefore contribute to the modulation of numerous health outcomes associated with breastfeeding and offer potential innovative therapeutic approaches.

## 6. MicroRNAs

Breast milk is one of the richest sources of microRNAs, which are small non-coding RNAs regulating gene expression at the post-transcriptional level.

MicroRNAs are covered and carried through the bloodstream by extracellular vesicles. These vesicles are released by various cells, including lactocytes, and contribute to intercellular communication promoting cellular crosstalk. In particular, exosomes are a subtype of extracellular vesicles composed of an endosomal route and are typically 30–150 nanometers in diameter. On the other hand, if the vesicles are 150 and 1000 nanometers in diameter, they are called micro-vesicles [79]. It has been hypothesized that the microRNAs present in breast milk, thanks to the extracellular vesicles, could be resistant to the gastrointestinal tract degradation because of their resistance to RNase digestion and tolerance of low pH and, through the bloodstream, could reach all the target organs or could perform their function locally [80,81]. Among the most innovative findings is evidence of endogenous synthesis of human milk miRNAs within the human lactating mammary epithelium [82]. It should be remembered that the microRNAs present in breast milk could modulate the function of genes involved in numerous physiological processes, including energy metabolism, immunological functions and cognitive development, even if with not yet fully understood mechanisms [82]. Moreover, according to recent studies, microRNAs appear to be involved in regulating genes associated with lipid metabolism at the post-transcriptional level [83]. A recent study by Carr et al. using an animal model evaluated that miRNAs found in breast milk were decreased in the serum of formula-fed infants, suggesting different circulating miRNA profiles based on neonatal diet [84].

Since miRNAs in breast milk were first described in 2007 [85], literature had focused on their possible physiological and therapeutic functions in cell proliferation, inflammation, immunomodulation and carcinogenesis. In the oncological field, miRNAs could play a role as onco-suppressors and oncogenes and, in specific types of cancer, they could be considered biomarkers and may represent useful diagnostic and therapeutic targets [86,87,88]. In addition, breast milk exosomes regulate inflammation and cell proliferation and, as explained in various recent studies, promote gut epithelial proliferation and intestinal stem cells activity, reducing NEC incidence in infants [89,90,91]. Furthermore, the recent proteomic analysis highlighted that breast milk exosomes revealed an immunomodulatory effect. Samuel et al. showed that colostrum’s exosomes were enriched in proteins involved in innate immune response, complement activation and inflammatory response and that exosomes in mature milk were enriched in proteins implicated in apoptosis [92]. Exosomes derived by stem cells have also been the subject of great interest from the scientific community.

## 7. Growth Factors and Hormones

Growth factors play an essential role among breast milk bioactive substances and have been extensively evaluated in the literature. Their primary function is to support the newborn’s growth through the proliferation and differentiation of their immature cells. Vascular endothelial growth factor (VEGF), hepatocyte growth factor (HGF), glucagon-like peptide-1 (GLP-1), epithelial growth factor (EGF) and insulin growth factors (IGFs) are amongst the most significant growth factors detected in breast milk [11]. In particular, the colostrum provides the highest concentrations to meet the increased postnatal needs, such as gut epithelium maturation, immune response and neurocognitive development [11]. Moreover, Patki et al. highlighted that some of these bioactive components, such as HGF and VEGF, are more represented in breast milk than in maternal serum, assuming a direct production by the mammary gland [93].

Recent studies investigated the role of various hormones in breast milk in regulating appetite, energy balance and fat mass deposition [10]. Amongst them, leptin, ghrelin, insulin growth factor 1 (IGF-1), adiponectin and insulin have the most significant influence on the infant’s growth and body composition and, as a result, on the risk of obesity in adulthood.

Discordant results were found regarding the correlation between leptin’s breast milk concentration and the risk of obesity later in life. Following Fields et al., leptin in breast milk has been inversely associated with fat mass deposition in newborns [94]. In addition, greater leptin and insulin concentrations have been associated with lower BMI-for-age z-scores [94,95]. These effects could be temporary since they are not confirmed after the first years of life [96]. On the other hand, Uysal et al. and Khodabakhshi et al. in their recent studies did not find any correlations between breast milk leptin concentrations and BMI of exclusively breastfed infants [97,98]. 

On the contrary, ghrelin and adiponectin, acting on the hypothalamus, stimulate hunger. Most studies have shown a higher concentration of ghrelin in breast milk than in serum in both newborns and mothers during the first months of life. However, inconsistent results have been obtained regarding the correlation between breast milk ghrelin and weight gain in infants [99]. Moreover, the most extensive cohort study found no association between adiponectin and infants’ body composition [96]. IGF-1 plays a primary role in cell proliferation and apoptosis inhibition. According to recent literature, infants with more significant weight gain consume breast milk with a higher level of IGF-1 [100]. However, the results in literature on the association between breast milk IGF-1 concentrations and infants’ weight gain are still discordant [10]. The inconsistency found with regards to some data might be due to the presence of several factors which can interfere with the infant’s body composition during the first years of life [93]. Therefore, further studies are needed to obtain more consistent results.

**Figure 3:**
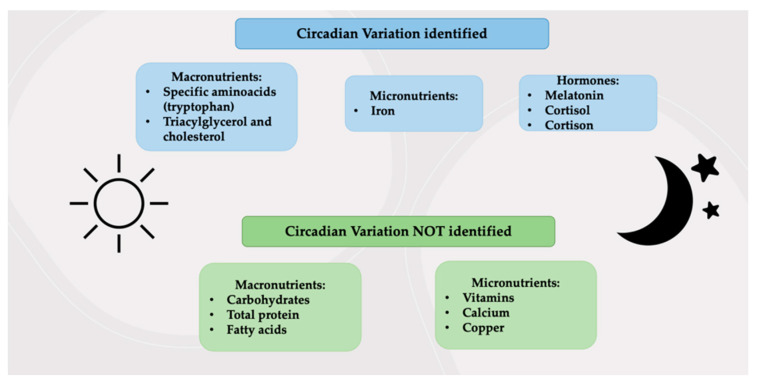
Components of breast milk affected or not by the circadian variation.

Recent studies have introduced an emerging field of interest known as chrono-nutrition, which is based on the relationship between circadian rhythm, metabolic health and temporal eating patterns [101]. The newborn’s circadian rhythm seems to be established by external factors such as light/dark exposure and the timing of feeding. At the same time, human milk components, especially the bioactive ones, exhibit a circadian variation of their concentrations [102]. Thus, the fluctuation within the composition of breast milk represents a mechanism by which information on time of day is transferred from the mother to the infant, facilitating the development of stable circadian rhythms and consequently the regulation of several fundamental activities and functions such as sleep, metabolism and hormones release [12]. According to these findings, human milk represents a powerful type of chrono-nutrition [12] (Figure 3).

## 8. Antioxidant Properties

Reactive oxygen species (ROS) participate in cellular signaling processes. However, ROS can damage cells due to their high oxidizing capacity. A powerful tool to neutralize these oxidative effects is the antioxidant system. However, if the precarious balance between ROS and antioxidants is lost, the result is oxidative stress. Birth denotes considerable oxidative stress, considering the transition from low-oxygen intrauterine to the high-oxygen extrauterine atmosphere [103]. Antioxidants can be classified as exogenous or endogenous. Endogenous antioxidants comprise specific enzymes (i.e., superoxide dismutase SOD, catalase or glutathione peroxidase GPx), small non-enzymatic molecules (i.e., glutathione GSH) or hormones such as melatonin [104]. Antioxidants are essential for newborn protection against disease [103]. Human milk has an antioxidant capacity. Accordingly, all of the compounds mentioned above have been found in it. The antioxidant properties of human milk are correlated both to exogenous food-derived antioxidants (i.e., polyphenols, carotenoids and vitamins and endogenous molecules, including enzymatic antioxidants such as catalase and GPx). However, it has to be taken into consideration that excessive antioxidants may be deleterious, and some may turn into pro-oxidant molecules under certain circumstances. Accordingly, the concentration of these antioxidants in formula milk does not always match breast milk, resulting in a much higher dose (Table 2) [105]. There is evidence that breast-milk-fed infants have less oxidative stress, evidenced by lower oxidative damage biomarkers than formula-fed infants [106]. Regarding non-enzymatic systems, GSH, a three amino acid peptide that engages in regenerating other antioxidants, such as vitamin C and E, to their active forms, has also been found in human milk [107]. Melatonin, as mentioned above, helps the newborn with circadian regulation. However, melatonin has also been confirmed to display protective antioxidant effects, acting both as a scavenger and as a stimulation factor for the expression of SOD, catalase and GPx [108]. Moreover, it has neuroprotection properties by modulating the neuroinflammatory pathways [109]. The antioxidant potential of breast milk is more powerful in colostrum compared to mature milk [110]. Its radical scavenging function decreases along the lactation period.

## 9. Conclusions

The scientific goals on understanding the beneficial effects of human milk on an infant’s growth and development are undoubtedly surprising. However, these significant achievements are not at all exhaustive. Beyond the nutritional value, the uniqueness of human milk is undoubtedly represented by its bioactive factors and their dynamic composition, which contribute to the infant’s growth, development and health [111]. However, the extreme complexity of the synergistic mechanisms responsible for breast milk’s numerous functional effects has not been fully clarified, thus leaving several points open. A better understanding of breast milk bioactivity may lead to new strategies in improving infants’ short- and long-term outcomes, including neurocognitive, gut and immune functions; the risk of early infections; and protection against overweight and obesity, hypertension, type 2 diabetes and atopic disease during adolescence and adulthood [112]. Understanding how human milk bioactive factors work may lead to the development of new clinical approaches and therapies, thus representing a relevant area of future research.

## Figures and Tables

**Table 1:** Most typically represented intestinal bacterial species based on the type of feeding. Expressed breast milk: EBM.

Type of Milk Feeding	Breastmilk	EBM	Formula Milk
Intestinal bacterial species	Bifidobacterium, Lactobacillus	Staphylococcaceae, Clostridiaceae, Pasteurellaceae	Clostridia

**Table 2:** Exogenous antioxidants in breast milk compared to formula milk. (Modified from Hanson et al., 2016.)

Antioxidant Compounds	Preterm Breastmilk	Term Breastmilk	Preterm Formula	Term Formula
α-carotene	7.7	3.6	0.51	1.40
β-carotene	49.1	13.7	71.1	63.9
Lycopene	66.1	11.9	1.5	5.8
Retinol	401.6	185.8	3086.2	911.8
α-tocopherol	5880.8	1381.9	20,109.1	13,360.2
γ-tocopherol	1207.1	622.8	6787.1	6561.6

Data are expressed as μg/L.

## Data Availability

Data sharing not applicable.

## References

[1] Mosca F., Giannì M.L. (2017). Human milk: Composition and health benefits. Pediatr. Med. Chir..

[2] Agostoni C., Braegger C., Decsi T., Kolacek S., Koletzko B., Michaelsen K.F., Mihatsch W., Moreno L.A., Puntis J., Shamir R. (2009). Breast-feeding: A commentary by the ESPGHAN Committee on Nutrition. J. Pediatr. Gastroenterol. Nutr..

[3] WHO (2018–2019). Protecting, Promoting, and Supporting Breastfeeding in Facilities Providing Maternity and Newborn Services: The Revised Baby-Friendly Hospital Initiative.

[4] American Academy of Pediatrics (2012). Breastfeeding and the use of human Milk. Pediatrics.

[5] Verduci E., Giannì M.L., Vizzari G., Vizzuso S., Cerasani J., Mosca F., Zuccotti G.V. (2021). The triad mother-breast milk-infant as predictor of future health: A narrative review. Nutrients.

[6] Granger C.L., Embleton N.D., Palmer J.M., Lamb C.A., Berrington J.E., Stewart C.J. (2021). Maternal breastmilk, infant gut microbiome and the impact on preterm infant health. Acta Paediatr..

[7] Leon D.A., Ronalds G. (2009). Breast-feeding influences on later life--cardiovascular disease. Adv. Exp. Med. Biol..

[8] Oddy W.H., Goldberg G., Prentice A., Prentice A., Filteau S., Simondon K. (2009). The long-term effects of breast-feeding on asthma and atopic disease. Breast-Feeding: Early Influences on Later Health.

[9] Carr L.E., Virmani M.D., Rosa F., Munblit D., Matazel K.S., Elolimy A.A., Yeruva L. (2021). Role of human milk bioactives on infants’ gut and immune health. Front. Immunol..

[10] Mazzocchi A., Giannì M.L., Morniroli D., Leone L., Roggero P., Agostoni C., De Cosmi V., Mosca F. (2019). Hormones in breast milk and effect on infants’ growth: A systematic review. Nutrients.

[11] Gila-Diaz A., Arribas S.M., Algara A., Martín-Cabrejas M.A., López de Pablo Á.L., Sáenz de Pipaón M., Ramiro-Cortijo D. (2019). A review of bioactive factors in human breastmilk: A focus on prematurity. Nutrients.

[12] Italianer M., Naninck E., Roelants J., Van Der Horst G., Reiss I., Goudoever J., Joosten K., Chaves I., Vermeulen M. (2020). Circadian variation in human milk composition, a systematic review. Nutrients.

[13] Vorbach C., Capecchi M.R., Penninger J.M. (2006). Evolution of the mammary gland from the innate immune system?. Bioessays.

[14] Schneider C., Illi M., Lötscher M., Wehrli M., Von Gunten S., Kaveri S.V., Bayry J. (2017). Isolation of antibodies from human plasma, saliva, breast milk, and gastrointestinal fluid. Natural Antibodies.

[15] Van de Perre P. (2003). Transfer of antibody via mother’s milk. Vaccine.

[16] Morniroli D., Consales A., Crippa B.L., Vizzari G., Ceroni F., Cerasani J., Colombo L., Mosca F., Giannì M.L. (2021). The antiviral properties of human milk: A multitude of defence tools from mother nature. Nutrients.

[17] Nolan L.S., Parks O.B., Good M. (2019). A review of the immunomodulating components of maternal breast milk and protection against necrotizing enterocolitis. Nutrients.

[18] Gopalakrishna K.P., Hand T.W. (2020). Influence of maternal milk on the neonatal intestinal microbiome. Nutrients.

[19] Donaldson G.P., Ladinsky M.S., Yu K.B., Sanders J.G., Yoo B.B., Chou W.-C., Conner M.E., Earl A.M., Knight R., Bjorkman P.J. (2018). Gut microbiota utilize immunoglobulin A for mucosal colonization. Science.

[20] Laouar A. (2020). Maternal leukocytes and infant immune programming during breastfeeding. Trends Immunol..

[21] Joseph C.L.M., Havstad S., Bobbitt K., Woodcroft K., Zoratti E.M., Nageotte C., Misiak R., Enberg R., Nicholas C., Ezell J.M. (2014). Transforming growth factor beta (TGFβ1) in breast milk and indicators of infant atopy in a birth cohort. Pediatr. Allergy Immunol..

[22] Bhatt H. (2021). Should COVID-19 mother breastfeed her newborn child? A literature review on the safety of breastfeeding for pregnant women with COVID-19. Curr. Nutr. Rep..

[23] Pace R.M., Williams J.E., Järvinen K.M., Belfort M.B., Pace C.D.W., Lackey K.A., Gogel A.C., Nguyen-Contant P., Kanagaiah P., Fitzgerald T. (2021). Characterization of SARS-CoV-2 RNA, antibodies, and neutralizing capacity in milk produced by women with COVID-19. mBio.

[24] Zhu F., Zozaya C., Zhou Q., De Castro C., Shah P.S. (2021). SARS-CoV-2 genome and antibodies in breastmilk: A systematic review and meta-analysis. Arch. Dis. Child Fetal Neonatal. Ed..

[25] Favara D.M., Ceron-Gutierrez M.L., Carnell G.W., Heeney J.L., Corrie P., Doffinger R. (2020). Detection of breastmilk antibodies targeting SARS-CoV-2 nucleocapsid, spike and receptor-binding-domain antigens. Emerg. Microbes Infect..

[26] Gray K.J., Bordt E.A., Atyeo C., Deriso E., Akinwunmi B., Young N., Baez A.M., Shook L.L., Cvrk D., James K. (2021). Coronavirus disease 2019 vaccine response in pregnant and lactating women: A cohort study. Am. J. Obstet. Gynecol..

[27] Cacho N.T., Lawrence R.M. (2017). Innate immunity and breast milk. Front. Immunol..

[28] Hackam D.J., Sodhi C.P. (2018). Toll-like receptor–mediated intestinal inflammatory imbalance in the pathogenesis of necrotizing enterocolitis. Cell. Mol. Gastroenterol. Hepatol..

[29] Hackam D.J., Sodhi C.P., Good M. (2019). New insights into necrotizing enterocolitis: From laboratory observation to personalized prevention and treatment. J. Pediatr. Surg..

[30] Kiełbasa A., Gadzała-Kopciuch R., Buszewski B. (2021). Cytokines-biogenesis and their role in human breast milk and determination. Int. J. Mol. Sci..

[31] Thai J.D., Gregory K.E. (2020). Bioactive factors in human breast milk attenuate intestinal inflammation during early life. Nutrients.

[32] Albenzio M., Santillo A., Stolfi I., Manzoni P., Iliceto A., Rinaldi M., Magaldi R. (2016). Lactoferrin levels in human milk after preterm and term delivery. Am. J. Perinatol..

[33] Pammi M., Gautham K.S. (2020). Enteral lactoferrin supplementation for prevention of sepsis and necrotizing enterocolitis in preterm infants. Cochrane Database Syst. Rev..

[34] Griffiths J., Jenkins P., Vargova M., Bowler U., Juszczak E., King A., Linsell L., Murray D., Partlett C., Patel M. (2019). Enteral lactoferrin supplementation for very preterm infants: A randomised placebo-controlled trial. Lancet.

[35] Yu J.C., Khodadadi H., Malik A., Davidson B., Da Silva E.L.S., Bhatia J., Hale V.L., Baban B. (2018). Innate immunity of neonates and infants. Front. Immunol..

[36] Coscia A., Bardanzellu F., Caboni E., Fanos V., Peroni D.G. (2021). When a neonate is born, so is a microbiota. Life.

[37] Padilha M., Danneskiold-Samsøe N.B., Brejnrod A., Hoffmann C., Cabral V.P., Iaucci J.D.M., Sales C.H., Fisberg R.M., Cortez R.V., Brix S. (2019). The human milk microbiota is modulated by maternal diet. Microorganisms.

[38] Fernández L., Rodríguez J.M., Ogra P.L., Walker W.A., Lönnerdal B. (2020). Human milk microbiota: Origin and potential uses. Nestlé Nutrition Institute Workshop Series.

[39] Pannaraj P.S., Li F., Cerini C., Bender J.M., Yang S., Rollie A., Adisetiyo H., Zabih S., Lincez P.J., Bittinger K. (2017). Association between breast milk bacterial communities and establishment and development of the infant gut microbiome. JAMA Pediatr..

[40] Bäckhed F., Roswall J., Peng Y., Feng Q., Jia H., Kovatcheva-Datchary P., Li Y., Xia Y., Xie H., Zhong H. (2015). Dynamics and stabilization of the human gut microbiome during the first year of life. Cell Host Microbe.

[41] Duranti S., Lugli G.A., Mancabelli L., Armanini F., Turroni F., James K., Ferretti P., Gorfer V., Ferrario C., Milani C. (2017). Maternal inheritance of bifidobacterial communities and bifidophages in infants through vertical transmission. Microbiome.

[42] Mohandas S., Pannaraj P.S., Ogra P.L., Walker W.A., Lönnerdal B. (2020). Beyond the bacterial microbiome: Virome of human milk and effects on the developing infant. Nestlé Nutrition Institute Workshop Series.

[43] Moossavi S., Azad M.B. (2020). Origins of human milk microbiota: New evidence and arising questions. Gut Microbes.

[44] Carrothers J.M., York M.A., Brooker S.L., Lackey K.A., Williams J.E., Shafii B., Price W.J., Settles M.L., McGuire M.A., McGuire M.K. (2015). Fecal microbial community structure is stable over time and related to variation in macronutrient and micronutrient intakes in lactating women. J. Nutr..

[45] Browne P.D., Aparicio M., Alba C., Hechler C., Beijers R., Rodríguez J.M., Fernández L., De Weerth C. (2019). Human milk microbiome and maternal postnatal psychosocial distress. Front. Microbiol..

[46] Currie J., Rossin-Slater M. (2015). Early-life origins of life-cycle well-being: Research and policy implications: Early-life origins of life-cycle well-being. J. Policy Anal. Manag..

[47] Darnton-Hill I., Nishida C., James W. (2004). A Life course approach to diet, nutrition and the prevention of chronic diseases. Public Health Nutr..

[48] Cabrera-Rubio R., Mira-Pascual L., Mira A., Collado M.C. (2016). Impact of mode of delivery on the milk microbiota composition of healthy women. J. Dev. Orig. Health Dis..

[49] Rodríguez J.M. (2014). The origin of human milk bacteria: Is there a bacterial entero-mammary pathway during late pregnancy and lactation?. Adv. Nutr..

[50] Hermansson H., Kumar H., Collado M.C., Salminen S., Isolauri E., Rautava S. (2019). Breast milk microbiota is shaped by mode of delivery and intrapartum antibiotic exposure. Front. Nutr..

[51] Chong C., Bloomfield F., O’Sullivan J. (2018). Factors affecting gastrointestinal microbiome development in neonates. Nutrients.

[52] Kumar H., Du Toit E., Kulkarni A., Aakko J., Linderborg K.M., Zhang Y., Nicol M.P., Isolauri E., Yang B., Collado M.C. (2016). Distinct patterns in human milk microbiota and fatty acid profiles across specific geographic locations. Front. Microbiol..

[53] Ford S.L., Lohmann P., Preidis G.A., Gordon P.S., O’Donnell A., Hagan J., Venkatachalam A., Balderas M., Luna R.A., Hair A.B. (2019). Improved feeding tolerance and growth are linked to increased gut microbial community diversity in very-low-birth-weight infants fed mother’s own milk compared with donor breast milk. Am. J. Clin. Nutr..

[54] Biagi E., Aceti A., Quercia S., Beghetti I., Rampelli S., Turroni S., Soverini M., Zambrini A.V., Faldella G., Candela M. (2018). Microbial community dynamics in mother’s milk and infant’s mouth and gut in moderately preterm infants. Front. Microbiol..

[55] Beghetti I., Biagi E., Martini S., Brigidi P., Corvaglia L., Aceti A. (2019). Human milk’s hidden gift: Implications of the milk microbiome for preterm infants’ health. Nutrients.

[56] Weaver G., Bertino E., Gebauer C., Grovslien A., Mileusnic-Milenovic R., Arslanoglu S., Barnett D., Boquien C.-Y., Buffin R., Gaya A. (2019). Recommendations for the establishment and operation of human milk banks in Europe: A consensus statement from the European Milk Bank Association (EMBA). Front. Pediatr..

[57] Picaud J.-C., Buffin R. (2017). Human milk—Treatment and quality of banked human milk. Clin. Perinatol..

[58] Zielinska M., Hamulka J., Grabowicz-Chądrzyńska I., Bryś J., Wesolowska A. (2019). Association between breastmilk LC PUFA, carotenoids and psychomotor development of exclusively breastfed infants. Int. J. Environ. Res. Public Health.

[59] Dogaru C.M., Nyffenegger D., Pescatore A.M., Spycher B.D., Kuehni C.E. (2014). Breastfeeding and childhood asthma: Systematic review and meta-analysis. Am. J. Epidemiol..

[60] Klopp A., Vehling L., Becker A.B., Subbarao P., Mandhane P.J., Turvey S.E., Lefebvre D.L., Sears M.R., Azad M.B., Daley D. (2017). Modes of infant feeding and the risk of childhood asthma: A prospective birth cohort study. J. Pediatr..

[61] Azad M.B., Vehling L., Lu Z., Dai D., Subbarao P., Becker A.B., Mandhane P.J., Turvey S.E., Lefebvre D.L., Sears M.R. (2017). Breastfeeding, maternal asthma and wheezing in the first year of life: A longitudinal birth cohort study. Eur. Respir. J..

[62] Wesolowska A., Sinkiewicz-Darol E., Barbarska O., Bernatowicz-Lojko U., Borszewska-Kornacka M.K., Van Goudoever J.B. (2019). Innovative techniques of processing human milk to preserve key components. Nutrients.

[63] Walsh C., Lane J.A., Van Sinderen D., Hickey R.M. (2020). Human milk oligosaccharides: Shaping the infant gut microbiota and supporting health. J. Funct. Foods.

[64] Lyons K.E., Ryan C.A., Dempsey E.M., Ross R.P., Stanton C. (2020). breast milk, a source of beneficial microbes and associated benefits for infant health. Nutrients.

[65] Morniroli D., Giannì M.L., Consales A., Pietrasanta C., Mosca F. (2020). Human sialome and coronavirus disease-2019 (COVID-19) pandemic: An understated correlation?. Front. Immunol..

[66] Lis-Kuberka J., Orczyk-Pawiłowicz M. (2019). Sialylated oligosaccharides and glycoconjugates of human milk. The impact on infant and newborn protection, development and well-being. Nutrients.

[67] Hegar B., Wibowo Y., Basrowi R.W., Ranuh R.G., Sudarmo S.M., Munasir Z., Atthiyah A.F., Widodo A.D., Supriatmo S., Kadim M. (2019). The role of two human milk oligosaccharides, 2′-fucosyllactose and lacto-N-neotetraose, in infant nutrition. Pediatr. Gastroenterol. Hepatol. Nutr..

[68] Lefebvre G., Shevlyakova M., Charpagne A., Marquis J., Vogel M., Kirsten T., Kiess W., Austin S., Sprenger N., Binia A. (2020). time of lactation and maternal fucosyltransferase genetic polymorphisms determine the variability in human milk oligosaccharides. Front. Nutr..

[69] Bode L., Ogra P.L., Walker W.A., Lönnerdal B. (2020). Human milk oligosaccharides: Structure and functions. Nestlé Nutrition Institute Workshop Series.

[70] Wise A., Robertson B., Choudhury B., Rautava S., Isolauri E., Salminen S., Bode L. (2018). Infants are exposed to human milk oligosaccharides already in utero. Front. Pediatr..

[71] Austin S., De Castro C.A., Sprenger N., Binia A., Affolter M., Garcia-Rodenas C.L., Beauport L., Tolsa J.-F., Fumeaux C.J.F. (2019). Human milk oligosaccharides in the milk of mothers delivering term versus preterm infants. Nutrients.

[72] Lu J., Claud E.C. (2019). Connection between gut microbiome and brain development in preterm infants. Dev. Psychobiol..

[73] Vázquez E., Barranco A., Ramírez M., Gruart A., Delgado-García J.M., Martínez-Lara E., Blanco S., Martín M.J., Castanys E., Buck R. (2015). Effects of a human milk oligosaccharide, 2′-fucosyllactose, on hippocampal long-term potentiation and learning capabilities in rodents. J. Nutr. Biochem..

[74] Saben J.L., Sims C.R., Abraham A., Bode L., Andres A. (2021). Human milk oligosaccharide concentrations and infant intakes are associated with maternal overweight and obesity and predict infant growth. Nutrients.

[75] Morozov V., Hansman G., Hanisch F.-G., Schroten H., Kunz C. (2018). Human milk oligosaccharides as promising antivirals. Mol. Nutr. Food Res..

[76] Ramani S., Stewart C.J., Laucirica D.R., Ajami N.J., Robertson B., Autran C.A., Shinge D., Rani S., Anandan S., Hu L. (2018). Human milk oligosaccharides, milk microbiome and infant gut microbiome modulate neonatal rotavirus infection. Nat. Commun..

[77] Valverde-Villegas J.M., Durand M., Bedin A.-S., Rutagwera D., Kankasa C., Tuaillon E., Nagot N., Vande Perre P., Molès J.-P. (2020). Large stem/progenitor-like cell subsets can also be identified in the CD45^−^ and CD45^+^/high populations in early human milk. J. Hum. Lact..

[78] Kaingade P., Somasundaram I., Sharma A., Patel D., Marappagounder D. (2017). Cellular components, including stem-like cells, of preterm mother’s mature milk as compared with those in her colostrum: A pilot study. Breastfeed. Med..

[79] Melnik B.C., Schmitz G. (2017). MicroRNAs: Milk’s epigenetic regulators. Best Pract. Res. Clin. Endocrinol. Metab..

[80] Record M. (2014). Intercellular communication by exosomes in placenta: A possible role in cell fusion?. Placenta.

[81] Zhou Q., Li M., Wang X., Li Q., Wang T., Zhu Q., Zhou X., Wang X., Gao X., Li X. (2012). Immune-related microRNAs are abundant in breast milk exosomes. Int. J. Biol. Sci..

[82] Alsaweed M., Hartmann P., Geddes D., Kakulas F. (2015). MicroRNAs in breastmilk and the lactating breast: Potential immunoprotectors and developmental regulators for the infant and the mother. Int. J. Environ. Res. Public Health.

[83] Vickers K.C., Remaley A.T. (2012). Lipid-based carriers of microRNAs and intercellular communication. Curr. Opin. Lipidol..

[84] Carr L.E., Bowlin A.K., Elolimy A.A., Byrum S.D., Washam C.L., Randolph C.E., MacLeod S.L., Yeruva L. (2020). Neonatal diet impacts circulatory miRNA profile in a porcine model. Front. Immunol..

[85] Admyre C., Johansson S.M., Qazi K.R., Filén J.-J., Lahesmaa R., Norman M., Neve E.P.A., Scheynius A., Gabrielsson S. (2007). Exosomes with immune modulatory features are present in human breast milk. J. Immunol..

[86] Reif S., Shiff Y.E., Golan-Gerstl R. (2019). Milk-derived exosomes (MDEs) have a different biological effect on normal fetal colon epithelial cells compared to colon tumor cells in a miRNA-dependent manner. J. Transl. Med..

[87] Mollaei H., Safaralizadeh R., Rostami Z. (2019). MicroRNA replacement therapy in cancer. J. Cell Physiol..

[88] Ling H., Fabbri M., Calin G.A. (2013). MicroRNAs and other non-coding RNAs as targets for anticancer drug development. Nat. Rev. Drug Discov..

[89] Hock A., Miyake H., Li B., Lee C., Ermini L., Koike Y., Chen Y., Määttänen P., Zani A., Pierro A. (2017). Breast milk-derived exosomes promote intestinal epithelial cell growth. J. Pediatr. Surg..

[90] Cortez J., Makker K., Kraemer D.F., Neu J., Sharma R., Hudak M.L. (2018). Maternal milk feedings reduce sepsis, necrotizing enterocolitis and improve outcomes of premature infants. J. Perinatol..

[91] Martin C., Patel M., Williams S., Arora H., Sims B. (2018). Human breast milk-derived exosomes attenuate cell death in intestinal epithelial cells. Innate Immun..

[92] Samuel T.M., Binia A., De Castro C.A., Thakkar S.K., Billeaud C., Agosti M., Al-Jashi I., Costeira M.J., Marchini G., Martínez-Costa C. (2019). Impact of maternal characteristics on human milk oligosaccharide composition over the first 4 months of lactation in a cohort of healthy european mothers. Sci. Rep..

[93] Patki S., Patki U., Patil R., Indumathi S., Kaingade P., Bulbule A., Nikam A., Pishte A. (2012). Comparison of the levels of the growth factors in umbilical cord serum and human milk and its clinical significance. Cytokine.

[94] Fields D.A., Demerath E.W. (2012). Relationship of insulin, glucose, leptin, IL-6 and TNF-α in human breast milk with infant growth and body composition: Analytes in human breast-milk. Pediatr. Obes..

[95] Alderete T.L., Autran C., Brekke B.E., Knight R., Bode L., Goran M.I., Fields D.A. (2015). Associations between human milk oligosaccharides and infant body composition in the first 6 mo of life. Am. J. Clin. Nutr..

[96] Chan D., Goruk S., Becker A.B., Subbarao P., Mandhane P.J., Turvey S.E., Lefebvre D., Sears M.R., Field C.J., Azad M.B. (2018). Adiponectin, leptin and insulin in breast milk: Associations with maternal characteristics and infant body composition in the first year of life. Int. J. Obes..

[97] Uysal F., Önal E., Aral Y., Adam B., Dilmen U., Ardiçolu Y. (2002). Breast milk leptin: Its relationship to maternal and infant adiposity. Clin. Nutr..

[98] Khodabakhshi A., Ghayour-Mobarhan M., Rooki H., Vakili R., Hashemy S.I., Mirhafez S.R., Shakeri M.-T., Kashanifar R., Pourbafarani R., Mirzaei H. (2014). Comparative measurement of ghrelin, leptin, adiponectin, EGF and IGF-1 in breast milk of mothers with overweight/obese and normal-weight infants. Eur. J. Clin. Nutr..

[99] Cesur G., Ozguner F., Yilmaz N., Dundar B. (2012). The relationship between ghrelin and adiponectin levels in breast milk and infant serum and growth of infants during early postnatal life. J. Physiol. Sci..

[100] Kon I.Y., Shilina N.M., Gmoshinskaya M.V., Ivanushkina T.A. (2014). The study of breast milk IGF-1, leptin, ghrelin and adiponectin levels as possible reasons of high weight gain in breast-fed infants. Ann. Nutr. Metab..

[101] Flanagan A., Bechtold D.A., Pot G.K., Johnston J.D. (2021). Chrono-nutrition: From molecular and neuronal mechanisms to human epidemiology and timed feeding patterns. J. Neurochem..

[102] Christ E., Korf H.-W., Von Gall C. (2012). When does it start ticking? Ontogenetic development of the mammalian circadian system. Progress in Brain Research.

[103] Mutinati M., Pantaleo M., Roncetti M., Piccinno M., Rizzo A., Sciorsci R. (2014). Oxidative stress in neonatology. A review. Reprod. Domest. Anim..

[104] Aceti A., Beghetti I., Martini S., Faldella G., Corvaglia L. (2018). Oxidative stress and necrotizing enterocolitis: Pathogenetic mechanisms, opportunities for intervention, and role of human milk. Oxid. Med. Cell. Longev..

[105] Silvestre D., Miranda M., Muriach M., Almansa I., Jareo E., Romero F.J. (2008). Antioxidant capacity of human milk: Effect of thermal conditions for the pasteurization. Acta Paediatr..

[106] Friel J.K., Martin S.M., Langdon M., Herzberg G.R., Buettner G.R. (2002). Milk from mothers of both premature and full-term infants provides better antioxidant protection than does infant formula. Pediatr. Res..

[107] Ballatori N., Krance S.M., Notenboom S., Shi S., Tieu K., Hammond C.L. (2009). Glutathione dysregulation and the etiology and progression of human diseases. Biol. Chem..

[108] Reiter R., Tan D.-X., Cabrera J., D’Arpa D., Sainz R.M., Mayo J., Ramos S. (1999). The oxidant/antioxidant network: Role of melatonin. Neurosignals.

[109] Colella M., Biran V., Baud O. (2016). Melatonin and the newborn brain. Early Hum. Dev..

[110] Quiles J.L., Ochoa J.J., Ramirez-Tortosa M.C., Linde J., Bompadre S., Battino M., Narbona E., Maldonado J., Mataix J. (2006). Coenzyme Q concentration and total antioxidant capacity of human milk at different stages of lactation in mothers of preterm and full-term infants. Free Radic. Res..

[111] Witkowska-Zimny M., Kaminska-El-Hassan E. (2017). Cells of human breast milk. Cell. Mol. Biol. Lett..

[112] Hill D.R., Newburg D.S. (2015). Clinical applications of bioactive milk components. Nutr. Rev..

